# Pupil Size in Spider Eyes Is Linked to Post-Ecdysal Lens Growth

**DOI:** 10.1371/journal.pone.0015838

**Published:** 2010-12-31

**Authors:** Lisa M. Fenk, Karin Heidlmayr, Philipp Lindner, Axel Schmid

**Affiliations:** Department of Neurobiology, Faculty of Life Sciences, University of Vienna, Vienna, Austria; Dalhousie University, Canada

## Abstract

In this study we describe a distinctive pigment ring that appears in spider eyes after ecdysis and successively decreases in size in the days thereafter. Although pigment stops in spider eyes are well known, size variability is, to our knowledge, reported here for the first time. Representative species from three families (Ctenidae, Sparassidae and Lycosidae) are investigated and, for one of these species (*Cupiennius salei*, Ctenidae), the progressive increase in pupil diameter is monitored. In this species the pupil occupies only a fourth of the total projected lens surface after ecdysis and reaches its final size after approximately ten days. MicroCT images suggest that the decrease of the pigment ring is linked to the growth of the corneal lens after ecdysis. The pigment rings might improve vision in the immature eye by shielding light rays that would otherwise enter the eye via peripheral regions of the cornea, beside the growing crystalline lens.

## Introduction

Most spiders have eight simple eyes that can be divided into two different classes according to structural and functional differences. The anterior median eyes (AM eyes) are referred to as principal eyes. This eye pair points forward and the retinae can usually be moved by a varying number of eye muscles. AM eyes are everted eyes with the light absorbing segment of the photoreceptors turned towards the incident light. The other eye pairs – the posterior-median (PM), the posterior lateral (PL) and anterior-lateral (AL) eyes – are referred to as secondary eyes and can cover various fields of view. Their retinae cannot be moved and the photoreceptor segment bearing the microvilli is turned away from the incident light. The secondary eyes of most nocturnal spiders are equipped with a light-reflecting tapetum. For reviews dealing with spider eyes see e.g. [1], [2].

Pigment rings restrict the aperture in spider eyes. This iris, consisting of pigment cells situated between the rear surface of the cornea and the glassbody, was recognized in the early 19^th^ century [3]. We will refer to the opening left by the pigment ring as “pupil”. The diameter of the pupil in different spider species has been determined in numerous studies and was, implicitly or explicitly, assumed to be constant (e.g. [4], [5], [6], [7], [8], [9]). The extent or absence of the pigment stop in jumping spiders has been shown to be linked to habitat illumination: species observed in shaded forest habitats lack significant pigment stops whereas species living in sunny habitats show extensive pigment rings that can reduce the light flux into the eye by 50% [7].

We first observed variable pigment rings in *Cupiennius salei* (Ctenidae). The pupil's initial size and its successive post-ecdysal growth were determined for three groups of different ages. We hypothesized this process to have a causal connection to the maturation of the eyes after ecdysis and therefore expected post-ecdysal pigment-rings to be found in other spider families as well.

## Materials and Methods

### Post-ecdysal pupil size and its subsequent increase in *Cupiennius salei*

The nocturnal hunting spider *Cupiennius salei* is common in Central America. The animals used in this study were bred separately in glass jars (5 liter) in a greenhouse at 12 h:12 h day:night cycle and were fed *Calliphora sp*. once a week. The temperature (15–28°C) and relative humidity (70–80%) resembled the natural conditions found in *C. salei*'s habitat.

*Cupiennius salei* molts 11 times before it reaches adulthood [10], with the first instar outside the eggsac having a body size of about 2 mm and adults reaching a body length of up to 50 mm with a leg span of more than 120 mm. Under laboratory conditions the development is usually completed after one year, and the total life span is in the order of two years. Nine juvenile *Cupiennius salei* were selected for our experiments, forming three classes of spiders of different ages (five, seven and nine months respectively).

In order to determine the moment of ecdysis for each spider, a web camera was positioned in front of the glass jars containing the individual animals and was set to take a picture every five minutes. For the functioning of the camera, the light intensity at night time had to be slightly increased. The characteristic molting positions of the spiders, as described by Melchers [10], could easily be detected in the photos: *Cupiennius* prepares for ecdysis by attaching itself horizontally to a thread with its dorsal side pointing downwards. After the cuticle of the carapax has opened, the pedipalps, the legs, and finally the opisthosoma are extracted from the integument. Once ecdysis is completed, spiders perform characteristic leg movements that prevent the cuticle in the joint region from hardening [10].

After ecdysis the size of the pupil was measured. The animals were anaesthetized with CO_2_, or cooled down, and subsequently tethered onto a wooden spherical cap. The cap's base had a diameter of 105 mm and the spiders' legs could be attached to the cap with a piece of Parafilm without being bent. A hole in the Parafilm strip left the prosoma and opisthosoma free (see also [11]). The cap was connected to a magnetic stand by means of a ball bearing. By rotating the cap each eye could then be positioned in the horizontal plane under a reflected-light microscope to be photographed (Nikon DS-U1, Adaptors: Nikon Digital Sight DS-U1 and Camera Adaptor CMA-D2, Tokyo, Japan).

The eyes of each spider (left and right AM; left and right PM) were photographed once before ecdysis, and then daily during the first week and every second day during the second week. The lens and pupil diameters were measured using the program Lucia General 5.10 (Laboratory Imaging, Prague, Czech Republic). Both the lens and the pupil have a rather circular shape and the diameters were determined via an approximated circle calculated from a varying number of points that were placed by hand on the outer edge of the pupil and the lens on the images.

The uncertainty of the pupil diameters was estimated by the standard deviation calculated for the diameter of a given lens measured on the different photographs. It was found to be in the order of 5–10 µm, corresponding to roughly 1% of the lens diameters.

### Variable pupil size in other spider families

Two other spider species were examined for variable pigment rings: *Lycosa tarentula* (Lycosidae), belonging, as well as *Cupiennius*, to the superfamily of Lycosoidea, and *Heteropoda venatoria* (Sparassidae) belonging to the group Dionycha [12]. Five 4-month-old *H. venatoria* and one *L. tarentula*, that was just before its penultimate ecdysis, were kept in our laboratory under a 12 h:12 h day:night cycle. The *Heteropoda* spiderlings were placed in small glass jars (0.4 liter) and the *Lycosa* in a plastic terrarium (11 liter). The spiders were fed either flies (*Calliphora* sp.) or crickets (*Acheta domesticus*) once a week.

Since the objective here was only to document the variability of the pupil size, we did not determine the precise moment of ecdysis, but instead checked for exuviae on a daily basis.

One day after ecdysis and once again several days later the spiders were tethered and photographed as described above for *Cupiennius salei*.

### The function of the pigment stop

We tested the hypothesis that the pigment ring is linked to the growth of the corneal lens after ecdysis using X-ray microtomography (microCT) imaging of the cephalothoraxes of two *Cupiennius salei* – one spider was fixed 9 hours after ecdysis and the other 9 days after ecdysis. Both spiders were photographed, as described above, before they were prepared for the scans. The preparation followed the methodology proposed by Metscher [13], [14]: The samples were fixed in Bouin's solution which was washed out two days later with 70% ethanol. After dehydration in 100% ethanol the specimens were stained overnight with iodine (1% I_2_ in 100% ethanol). For the scans the samples were placed in small polypropylene tubes filled with 100% ethanol.

Both spiders were scanned with 66 kV and 133 µA at a 4.3 fold optical magnification (Xradia MicroXCT, Pleasanton, California), resulting in a projection image pixel size of 2.5 µm for the spider fixed 9 days after ecdysis and 2.0 µm for the other, slightly smaller, spider. Images were reconstructed with 2×2 pixel binning to reduce noise, resulting in final voxel sizes of 5.0 µm and 4.0 µm. Using the Xradia viewer software (TXM 3DViewer), virtual sections at any orientation through the reconstructed eyes could be viewed.

## Results

### Post-ecdysal pupil size and its subsequent increase in *Cupiennius salei*

**Figure 1:**
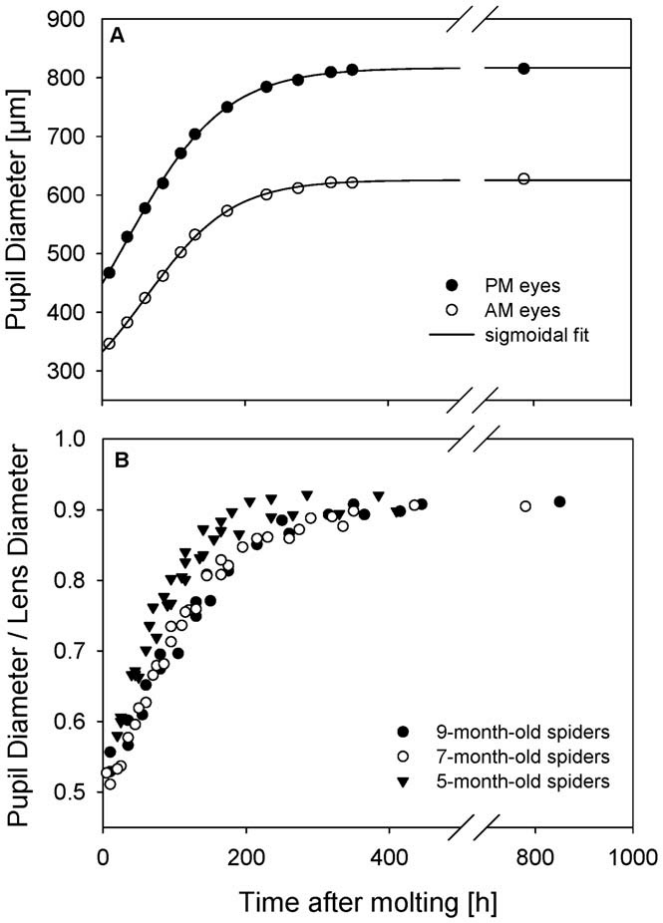
The pupil size as a function of time. (A) The mean pupil diameters of the PM and AM eyes for a 7-month-old spider as a function of the time after ecdysis. The data were fitted using *d* = *d_f_* –*a*/(1+exp((*t*–*t_0_*)/*b*)), where *d* is the pupil diameter and *d_f_* is its asymptotic value for large times *t*. PM eyes (r^2^ = 0.9993): *d_f_* = 817 µm, *a* = 600 µm, *t_0_* = 32 h, *b* = 69 h. AM eyes (r^2^ = 0.9997): *d_f_* = 626 µm, *a* = 400 µm, *t_0_* = 61 h, *b* = 60 h. (B) The pupil-to-lens ratios of the PM eyes of *Cupiennius salei*, for three different age groups as a function of the time after ecdysis.

The enlarged pigment rings were observed in both the principal and secondary eyes and at all three developmental stages examined (Fig. 1A, B). The mean diameters of the lenses of the AM and PM eyes for the three groups before and after ecdysis are given together with the initial pupil size estimated from extrapolating linear regressions calculated for a time *t*<130 hours after ecdysis in Table 1, 2. The initial pupil-to-lens ratio was found to be in the order of 0.5, i.e. only a fourth of the total projected lens surface is free from the pigment shield shortly after ecdysis.

**Table 1:** Lens and pupil diameters of the PM eyes.

	*d* of lens before ecdysis	*d* of lens after ecdysis	*d* of initial pupil
5-months (N = 4)	645	763	417
7-months (N = 3)	797	933	462
9-months (N = 2)	906	1009	527

The means of the diameter *d* (in µm) of the PM eye lenses for the three different age groups are given for N spiders before ecdysis and after ecdysis. The last column gives the values for *t* = 0 calculated from a linear regression for the first 130 hours after ecdysis as an estimate of the pupils' initial diameter.

**Table 2:** Lens and pupil diameters of the AM eyes.

	*d* of lens before ecdysis	*d* of lens after ecdysis	*d* of initial pupil
5-months (N = 4)	445	559	289
7-months (N = 3)	598	716	327
9-months (N = 2)	670	775	364

The means of the diameter *d* (in µm) of the AM eye lenses for the three different age groups are given for N spiders before ecdysis and after ecdysis. The last column gives the values for *t* = 0 calculated from a linear regression for the first 130 hours after ecdysis as an estimate of the pupils' initial diameter.

In the following days the pupil diameter *d* increased and the pupil-to-lens ratio converged towards a ratio of approximately 0.9. The growths of the PM and AM eye pupils of a 7-month-old spider with time *t* after molting are shown in Fig. 1A together with a sigmoidal fit (r^2^>0.999) following *d* = *d_f_* –*a*/(1+exp((*t*–*t_0_*)/*b*)). In both eye pairs 95% of the asymptotic values *d_f_* were reached after roughly 210 h.

The increase of the pupil diameter was found to be rather similar for the 7- and 9-month-old spiders, whereas the 5-month-old spiders seemed to have a slightly larger pupil-to-lens ratio after ecdysis which increased faster in size in the following days as compared to the older spiders (Fig. 1B).

### Variable pupil size in other spider families

**Figure 2:**
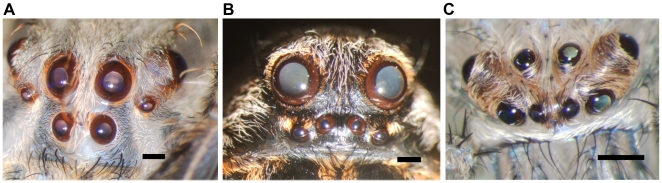
Photographs of freshly molted spiders. Portraits of *Cupiennius salei* (A), *Lycosa tarentula* (B) and *Heteropoda venatoria* (C) the day after ecdysis. In all three species the iris formed by the pigment ring is clearly visible and was observed to disappear in the days thereafter. Scale bars: 500 µm.

To test if a variable pupil size can be found in other spider species, we investigated *Lycosa tarentula* and *Heteropoda venatoria*. Both species indeed showed large pigment rings after ecdysis that disappeared almost entirely in the days thereafter. Pictures of *C. salei*, *L. tarentula* and *H. venatoria* one day after ecdysis are shown in Fig. 2.

### The function of the pigment stop

Slices through the reconstructed eyes of the spider scanned shortly after ecdysis show small lenses attached to the most central part of the corneal cap. The scan of the second spider, which was fixed 9 days after ecdysis, reveals a lens that has clearly increased in size compared to the cornea, and that now fills out more than the corneal cap.

**Figure 3:**
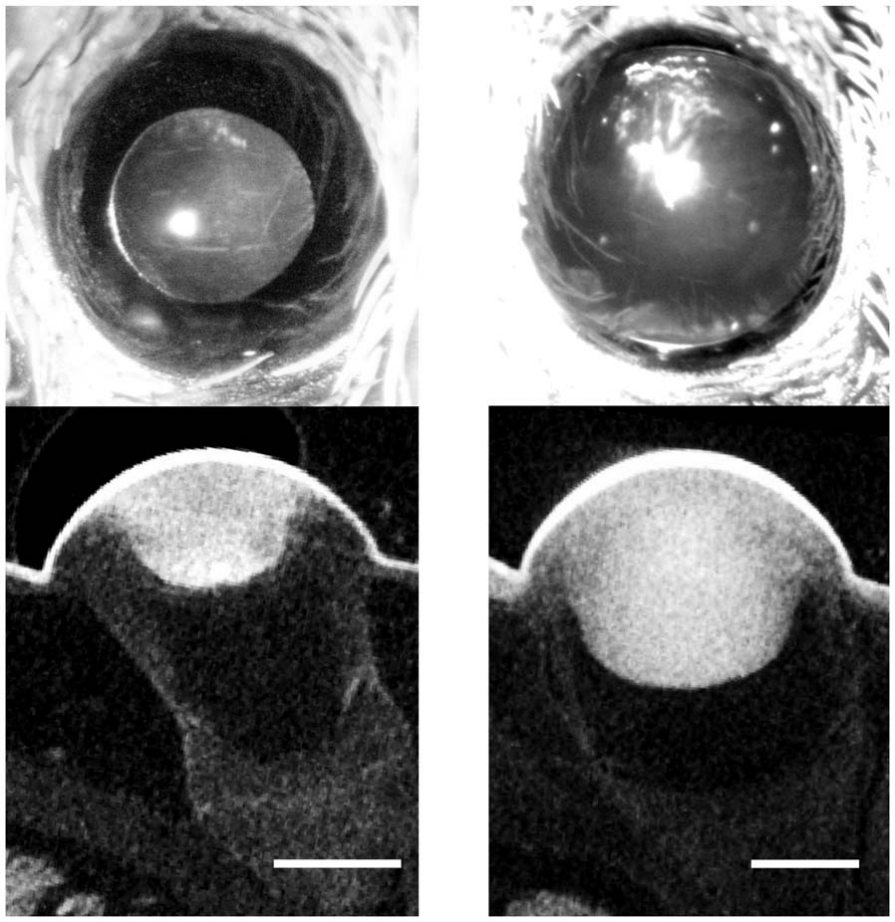
Comparison of micrographs and microCT scans. The upper part shows *in vivo* micrographs of a PM eye of a spider the day after ecdysis (left panel) and 9 days after ecdysis (right panel), taken just before the preparation of the spiders for the micro CT scans. The lower part shows sections through the reconstructed PM eyes. Scale bars: 200 µm.

A comparison of the *in vivo* micrographs with virtual slices in the plane of the PM eyes indicates a good matching of the part of the cornea covered by the lens and the pupil diameter (Fig. 3). Both the ratio of the pupil diameter to the lens diameter in the micrograph, and the ratio of the crystalline lens diameter at the corneal surface to the cornea diameter, is in the order of 0.55 in the post-ecdysal eye.

## Discussion

A detailed description of the molting of spider eyes is given by Wagner [15]. He recognized that the cuticular cornea and parts of the crystalline lens are formed before ecdysis and that the crystalline lens grows in the days thereafter. Browning reported similar findings for *Tegenaria* in a study that deals primarily with the structure of the cuticle before and after molting [16]. Our observations clearly confirm these descriptions. However, the crystalline lens seems to be initially restricted to the middle of the corneal cup (Fig. 3), which could not be derived from Wagner's and Browning's drawings. We assume that the more peripheral parts of the cornea, which are not covered by the growing lens, are shielded by the pigment rings. Wagner did certainly also observe the pigment rings, since he stated that in *Lycosa* only three quarters of the cornea are transparent. Interestingly, he reported the same for the cornea shed with the exuvia, whereas we found all parts of the eyes in the exuviae to be completely transparent. Moreover, Wagner did not mention any temporal variability of the pupil size and finally concluded that spiders probably have a limited field of view.

Future research on the visual systems of spiders should take the post-ecdysal development of the lens into account. Measurements of the aperture of the eyes are certainly difficult to compare without knowledge of the time since the last molt – at least for some spider species. On the other hand, large pigment rings might allow the identification of freshly molted individuals collected in their natural environment.

In kissing bugs (*Triatoma infestans*) Insausti and Lazzari [17] have specified a similar process, as we describe here for spiders. In *T. infestans* recently emerged adults show an elongated narrow pupil surrounded by pigment cells. The pupil widens during the following twenty days, and the change in pupil size corresponds well to the growth of the corneal lens. The authors suggest that the pigment rings are linked to the development of the ocelli [17]. Ocelli of insects can also be equipped with pupils that change size as a response to light stimuli as has been observed in two locust species [18].

In *Cupiennius salei* the lens in the mature eye produces an image of good quality on the retina as has been shown by Land and Barth using an ophthalmoscope [19]. A significant behavioral reaction of the spiders to moving gratings was measured down to spatial periods of 2° [20], which also indicates that the optical system is well developed in this nocturnal species.

We measured the radii of curvature of the corneal lens and the distance between the lens surfaces and the retina on the microCT sections for a *Cupiennius salei* fixed nine days after ecdysis. Considering these measurements, a homogeneous crystalline lens must be assumed to have a refractive index in the order of 1.67 to achieve enough refractive power to focus parallel rays on the retina. However, such a high refractive index is not common in biological materials and, as argued by Blest and Land, this suggests the existence of a graded refractive index, which could also correct for spherical aberrations [21]. Blest and Land investigated the large PM eyes of *Dinopis* and compared the radii of curvature to the focal length measured in excised lenses. Based on the radii of curvature the authors calculated the focal length as a function of the refractive index and found that a refractive index of 1.65–1.67 would be needed to achieve the focal length actually measured in *Dinopis*. The high apparent refractive index was taken as an indication for an inhomogeneous structure of the lens. This assumption was also corroborated by a less pronounced spherical aberration measured in these eyes than would be expected for a homogeneous lens [21]. If we assume this apparent refractive index for the eye of *Cupiennius salei* fixed nine hours after ecdysis, the calculations suggest that the image of an object at infinity is well focused on the retina for light rays passing through the lens and that the growing lens can already produce a sharp image on the retina.

The pigment rings might attenuate the impairment of vision which would result from peripheral light rays entering the eye beside the lens. In the absence of shielding pigment two posterior nodal distances must be expected in the immature eye: one for light passing only through the curved cornea, and a shorter one for light passing through the cornea plus the crystalline lens. If light rays were permitted to enter the eye beside the lens via the outer regions of the cornea, a second focal plane would be formed roughly 600 µm behind the retina, and additionally a strong spherical aberration should be expected. This would certainly severely degrade the quality of the image formed by the crystalline lens on the retina. But since the pigment rings restrict the aperture there are no reasons to believe that spatial resolution is much impaired after molting. However, the image on the retina must be expected to be less bright due to the smaller pupil and the accordingly higher F-number.

The total time in which spiders have to deal with immature eyes is by no means negligible and it might therefore be interesting to investigate in behavioral studies to what extent vision is available shortly after ecdysis.
